# Case Report: Improved axillary artery cannulation and extracorporeal membrane oxygenation bridging therapy for cardiogenic shock caused by Marfan syndrome combined with aortic dissection

**DOI:** 10.3389/fcvm.2026.1735797

**Published:** 2026-03-13

**Authors:** Fangfang Qiu, Lihan Luo, Junchao Huang, Jinshan Zhou

**Affiliations:** 1 Department of Critical Care Medicine, the Fourth Affiliated Hospital of School of Medicine, and International School of Medicine, International Institutes of Medicine, Zhejiang University, Yiwu, China; 2 Department of Cardio-Thoracic Surgery, The Fourth Affiliated Hospital of School of Medicine, and International School of Medicine, International Institutes of Medicine, Zhejiang University, Yiwu, China

**Keywords:** acute cardiogenic shock, aortic dissection, axillary artery catheterization, extracorporeal membrane oxygenation, Marfan syndrome

## Abstract

Marfan syndrome (MFS) is a congenital connective tissue disorder whose cardiovascular manifestations primarily affect the ascending aorta, presenting as ascending aortic aneurysm, aortic annulus dilation, aortic valve insufficiency, and aortic dissection ( 1). It has a poor prognosis and a high mortality rate.This article reports a case of a patient with MFS and acute Stanford type A aortic dissection. Hematoma compression led to left main coronary artery occlusion, resulting in a large myocardial infarction and severe low cardiac output syndrome. The patient was ultimately treated with a modified axillary artery cannulation for ECMO bridging therapy. This case study focuses on the advantages of ECMO cannulation using an axillary artery bridging graft over traditional peripheral and central cannulation, this cannulation technique offers advantages such as optimized hemodynamics, significantly reduced risk of limb ischemia, fewer infectious complications, and improved cerebral and coronary perfusion, making it one of the more ideal ECMO cannulation strategies. Despite initial success with ECMO support, the patient ultimately abandoned a left ventricular assist device (LVAD) and heart transplantation for financial reasons, resulting in clinical death. This case explores a modified axillary artery cannulation method to achieve antegrade blood flow, avoiding the complications of severe left ventricular afterload and left ventricular dilatation.

## Case

**Figure 1:**
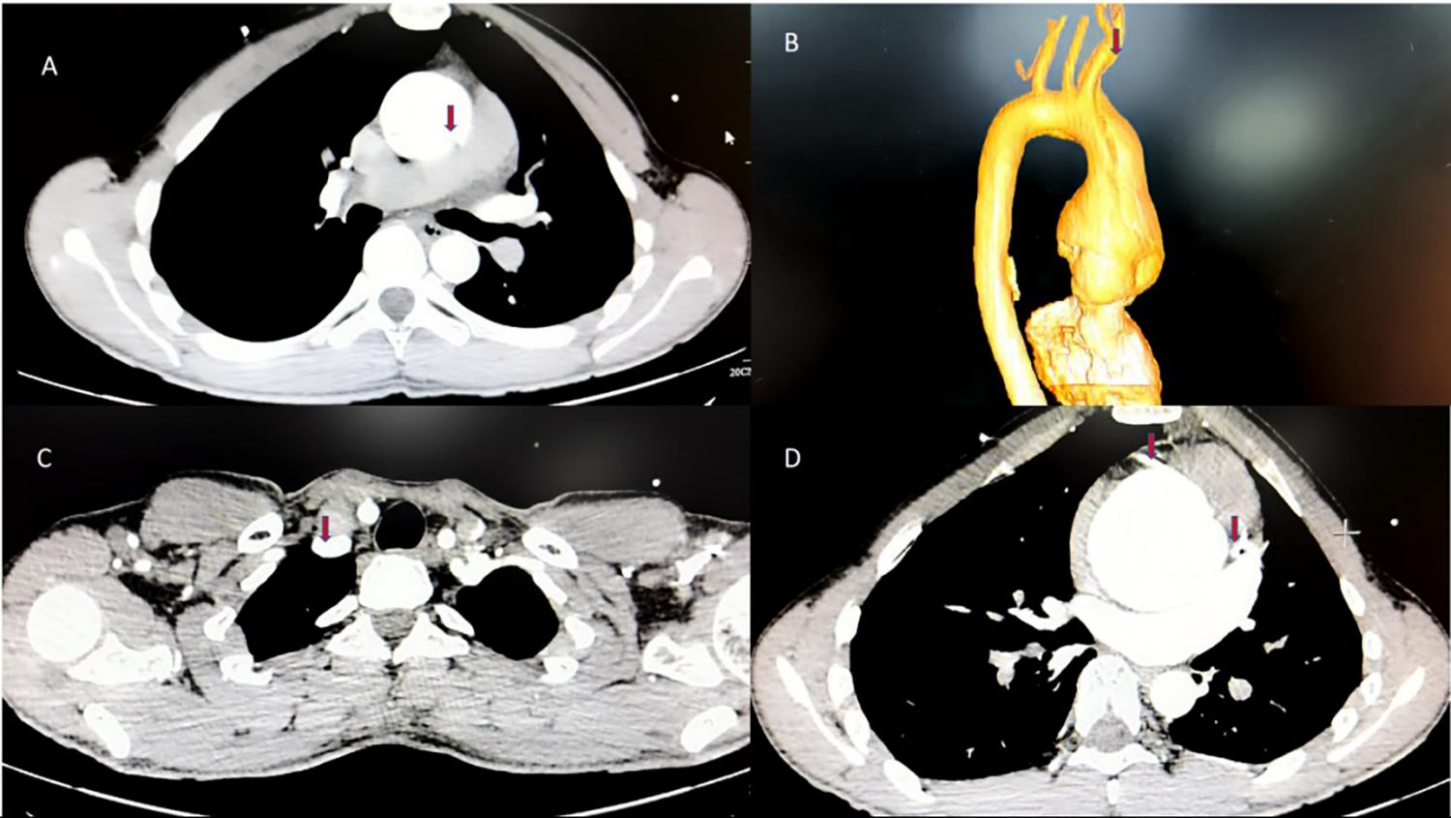
CTA of the thoracoabdominal aorta performed at the local hospital. **(A)** The patient presented with a “pigeon chest” deformity, and a significant aneurysmal dilation of the aortic root accompanied by AD, with a diameter of approximately 62 mm at its widest point; **(B)** the three-dimensional imaging showed an AD involving the proximal portion of the brachiocephalic artery; **(C)** the right subclavian artery is well-filled, and the dissection does not extend to it; **(D)** both left and right coronary arteries are well-filled, with no hematoma compression, ruling out the presence of acute myocardial infarction at this time.

This patient is a 19-year-old male who had been diagnosed with MFS. He currently weighs 60 kg, stands 185 cm tall, and has a body mass index (BMI) of 17.53. Three days ago, he experienced a transient, severe, tearing chest pain after work that lasted for several minutes before resolving, but he ignored it. Day 0, he experienced another episode of persistent, dull pain that was unbearable. Echocardiography at a local hospital revealed aneurysmal dilatation of the ascending aorta and moderate-to-severe aortic regurgitation, leading to the diagnosis of an aortic dissection (AD). Computed Tomography Angiography (CTA) of the thoracoabdominal aorta confirmed Stanford type A aortic dissection (Figure 1), at this time, both coronary arteries showed normal contrast agent opacification. He was transferred to our hospital for further treatment. The patient's mother, diagnosed with MFS, underwent two AD surgeries and passed away two years ago. His sister also has a history of MFS.

**Figure 2:**
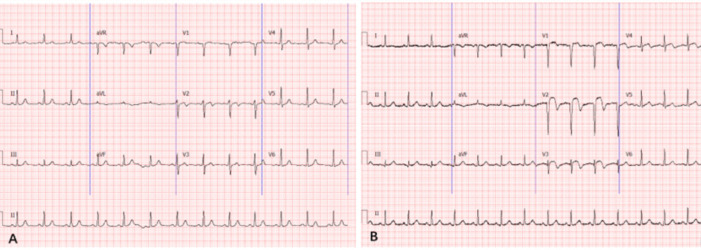
**(A)** Electrocardiogram shows sinus rhythm and no obvious abnormal changes; **(B)** electrocardiogram shows V1–V4 ST segment elevation, consistent with acute myocardial infarction.

Initial impressions of the patient at the pre-examination table were that he was conscious and aware, complaining of chest tightness and pain, with a respiratory rate (RR) of 18 bpm, a heart rate (HR) of 85 bpm, and a blood pressure (BP) of 82/62 mmHg, The chest wall showed a deformed bulge, consistent with pectus carinatum, bilateral lung sounds were clear., the apical impulse was located 0.5 cm medial to the left midclavicular line in the 5th intercostal space, a grade 3/6 diastolic murmur, described as a sighing sound, was heard in the aortic valve auscultation area, radiating to the apex, A2 < P2. no pericardial friction rub was heard in any valve auscultation area. the abdomen was soft, with no tenderness or rebound tenderness, bowel sounds were 3 times per min, there was no edema in the extremities, and pathological reflexes were not elicited. Complete echocardiography revealed: 1. Left ventricular enlargement, regional hypokinesis of the left ventricular anterior wall, decreased left ventricular systolic function, and a left ventricular ejection fraction (LVEF) of approximately 50%; 2. Aneurysmal dilatation of the ascending aorta and moderate to severe aortic regurgitation; 3. No significant pericardial effusion. An electrocardiogram (ECG) revealed sinus rhythm with no significant abnormalities (Figure 2). Blood test results are detailed in Table 1, Other blood tests showed no significant abnormalities. Due to poor circulation, symptomatic treatment was administered, including vasoactive medications to increase blood pressure, control ventricular rate, provide analgesia, and alleviate emotional distress. During the observation period in the emergency room, the dose of Norepinephrine (NE) was 0.89 ug/Kg/min. At this time, the vital signs were rechecked: HR 90 bpm; BP: left upper limb 89/67 (76) mmHg, right upper limb 85/62 (70) mmHg, right lower limb 105/82 (90) mmHg, left lower limb 108/84 (92) mmHg; oxygen was inhaled through the mask at 10 L/min, pulse oxygen saturation (SpO_2_) was 90%, and the symptoms of wet and cold extremities did not improve. 05:00 the patient experienced a rapid drop in blood pressure. With a norepinephrine dose of 1.11 ug/kg/min, the blood pressure was 70/54 (60) mmHg and the heart rate was 101 bpm. He then developed ventricular tachycardia. After treatment with amiodarone, he recovered to a transient sinus rhythm. A bedside electrocardiogram showed: V1-V ST segment elevation, consistent with acute myocardial infarction (Figure 2). At this time, Blood tests also showed abnormal results (Table 1). Comprehensive consideration of the patient's combined anterior wall acute myocardial infarction. Ventricular tachycardia soon recurred frequently, and amiodarone antiarrhythmic treatment was continued. Considering the rapid deterioration of the patient's circulatory function, another cardiac ultrasound examination showed: 1. Left ventricular (LV) enlargement 69 mm, diffuse left ventricular wall hypokinesia, decreased left ventricular systolic function and LVEF approximately 30%; 2. The ascending aorta was aneurysm dilated to 61.6 mm, with significant regurgitation at the aortic valve, suggesting severe aortic regurgitation. 3. The pericardial effusion had increased compared to before. At this point in his condition, we assessed that the aortic dissection had involved the left coronary artery, leading to severe low-output shock, and we planned to perform immediate surgery.

**Table 1:** The case shows important blood test indicators before surgery on Day 0.

Variables	03:00	05:00	Reference value	Unit
white blood cell (WBC)	26.2	24.0	3.5–9.5	*10^9^/L
Hemoglobin (Hb)	137	130	130–175	g/L
platelet (PLT)	284	257	125–350	*10^9^/L
Myoglobin (Mb)	152	>500	20–80	ng/mL
Cardiac troponin I (TnI)	0.114	>10	<0.014	ng/mL
Creatine kinase-MB (CK-MB)	22	388	<25	ng/mL
pH	7.258	7.287	—	—
Partial pressure of carbon dioxide (PaCO_2_)	46.9	39.7	36–44	mmHg
Partial pressure of oxygen (PaO_2_)	75	61	75–95	mmHg
Base wxcess (BE)	−6.3	−7.3	−3.0 to +3.0	mmol/L
Actual Bicarbonate (AB)	20.9	18.7	22.0–26.0	mmol/L
Lactic acid (Lac)	2.4	3.2	0.7–2.1	mmol/L
Noradrenaline (NE)	0.89	1.11	—	ug/Kg/min

**Figure 3:**
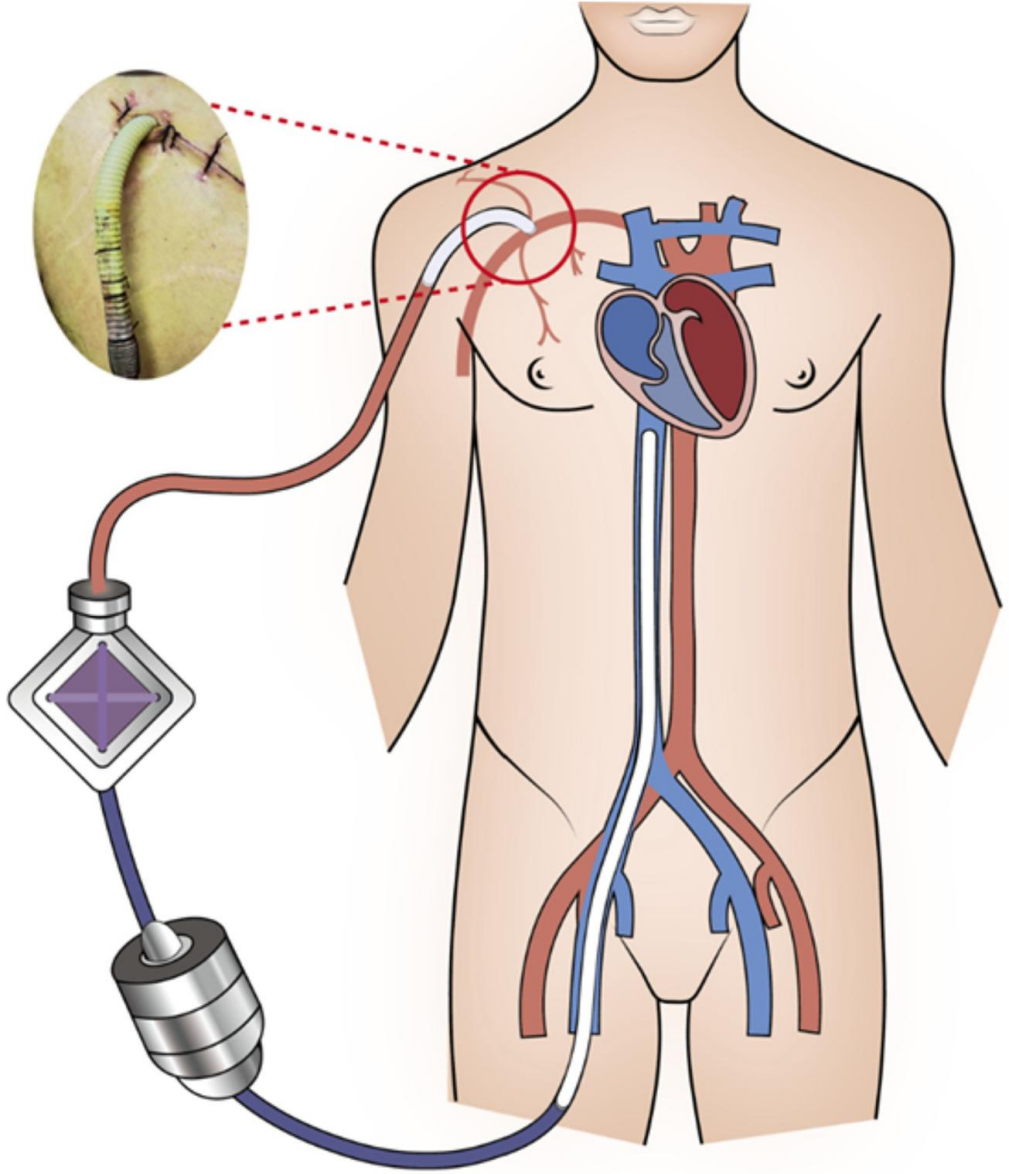
The ECMO perfusion tube is bridged to the axillary artery using an artificial blood vessel.

During surgery, after opening the pericardium, a large amount of bloody pericardial effusion was observed. The ascending aorta root was significantly thickened, approximately 6–7 cm wide. A hematoma compressed the main trunk of the left coronary artery. The dissection extended from the ascending aorta to the aortic arch and the proximal part of the brachiocephalic artery, with the brachiocephalic artery supplied by the true lumen. After establishing cardiopulmonary bypass and stopping the heart, the ascending aorta was incised with scissors. The dissection tear was located above the left coronary artery ostium, and the left coronary artery was involved in the dissection, with numerous thrombi in the medial layer. The left coronary artery was compressed, and the aortic valve was significantly dilated with insufficiency. The aortic valve annulus diameter was approximately 4 cm. A 25 mm Medtronic mechanical valve was then implanted after excising the aortic valve; the left internal mammary artery was harvested and grafted to the main trunk of the left coronary artery; considering the involvement of the medial layer dissection at the aortic arch, and although there were many intimal plaques, the intima was intact, so a partial ascending aorta resection with artificial graft replacement was performed. Artificial graft replacement was performed at the proximal dissection site of the brachiocephalic artery to ensure blood supply from the true lumen. After the completion of the above surgical steps, the heart gradually resumed beating automatically, but repeated attempts to stop extracorporeal circulation support revealed that the patient's left ventricular systolic function was extremely poor, with a pulse pressure difference that was always less than 20 mmHg. Even with the use of large doses of vasoactive drugs, normal blood pressure could not be maintained. Considering that the preoperative aortic dissection involved the left coronary artery, leading to left ventricular myocardial cell necrosis and left ventricular systolic function damage, it was decided to use Veno-Arterial Extracorporeal Membrane Oxygenation (VA-ECMO) instead to end the operation. Considering that central cannulation through the ascending aorta may complicate subsequent management, including increased risk of bleeding and infection, and that peripheral cannulation would increase left ventricular afterload on top of already poor cardiac function, further worsening left ventricular contraction, we considered the option of placing an additional graft on the right axillary artery. Through a subclavian incision, the distal end of the right axillary artery was exposed. A segment of 10 mm four-branched artificial blood vessel, approximately 10 cm in length, was harvested and anastomosed end-to-side with the subclavian artery and the ECMO perfusion cannula was inserted into the graft and secured with silk suture. Complete hemostasis was achieved, and the skin incision was sutured (Figure 3). This allowed the ECMO perfusion cannula to flow back into this vessel, avoiding the risks of central cannulation while reducing the increase in left ventricular afterload caused by peripheral cannulation. The initial ECMO settings were 2,594 r/min, flow rate 3.45 L/min, and gas flow rate 3 L/min. At this time, NE: 0.9 ug/Kg/min and epinephrine (Ad) 0.3 ug/Kg/min were combined to maintain blood pressure at 81/67 (72) mmHg, with the goal of maintaining a mean arterial pressure above 65 mmHg. After transfer to the ICU, blood pressure was monitored using the right radial artery. Due to the pulse pressure difference of approximately 10 mmHg, non-invasive blood pressure monitoring was not possible. Vasoactive drugs were gradually adjusted based on the right radial artery invasive blood pressure (MBP). NE and Ad were basically discontinued 6 h after the operation.

On the first day after admission, the patient's ECMO speed and flow rate did not change significantly, and blood gas analysis showed that metabolic acidosis did not improve, and lactate did not decrease progressively (Ph: 7.287, BE: −7.3 mmol/L, AB: 18.9 mmol/L, Lac: 8.7 mmol/L). The assessment suggested that the patient had insufficient tissue perfusion, so we reconstructed the left radial artery pressure measurement for comparison. At this time, the right side was 78/64 (69) mmHg, while the left side was 57/49 (52) mmHg, which was significantly lower than the minimum requirement for tissue perfusion. Therefore, we decided to use the left radial artery blood pressure monitoring as the basis, re-use vasoactive drugs to maintain MBP above 65 mmHg, and connect CRRT in parallel to the ECMO circuit to improve the internal environment and promote the excretion of metabolic products. The patient's metabolic acidosis in blood gas analysis gradually improved, and lactate showed a progressive downward trend (Ph: 7.404, BE: 0.8 mmol/L, AB On the second day after admission, the patient's vasoactive drug dosage remained untitrated (NE: 0.84 ug/kg/min, Ad: 0.25 ug/kg/min). Inflammatory markers at this time reported white blood cell (WBC) levels of 16.5 × 10^−9^/L, procalcitonin (PCT) levels of 16.9 ng/mL, and interleukin-6 (IL-6) levels of 837.8 pg/mL. Bedside ultrasound assessment indicated adequate blood flow and ruled out obstructive shock. Initial assessment suggested hypotonic shock, or septic shock, in addition to cardiogenic shock. Therefore, antibiotic therapy was upgraded to vancomycin combined with imipenem and cilastatin sodium. Following aggressive anti-infective treatment, the patient's circulation gradually improved, and the vasoactive drug dosage was gradually reduced. On the third day after admission, the patient's total bilirubin showed an abnormally elevated trend, and hemoglobin remained at a low level (59 g/L) despite intermittent red blood cell transfusion. Total Bilirubin (TB) was 175 μmol/L, of which indirect bilirubin (IB) was 110 μmol/L. Considering that the patient had mechanical hemolysis after prolonged and high-speed operation of the centrifugal pump, we adopted CRRT to accelerate the removal of metabolic waste while re-titrifying the flow rate to reduce the speed of the centrifugal pump head to reduce the shear force of the centrifugal pump on red blood cells. At this time, the ECMO parameters were: 2,200 r/min, flow rate 3.0 L/min, and gas flow rate 3.0 L/min, which effectively alleviated the occurrence of hemolysis, and continued to intermittently transfuse red blood cells to ensure the blood's oxygen-carrying capacity.

**Figure 4:**
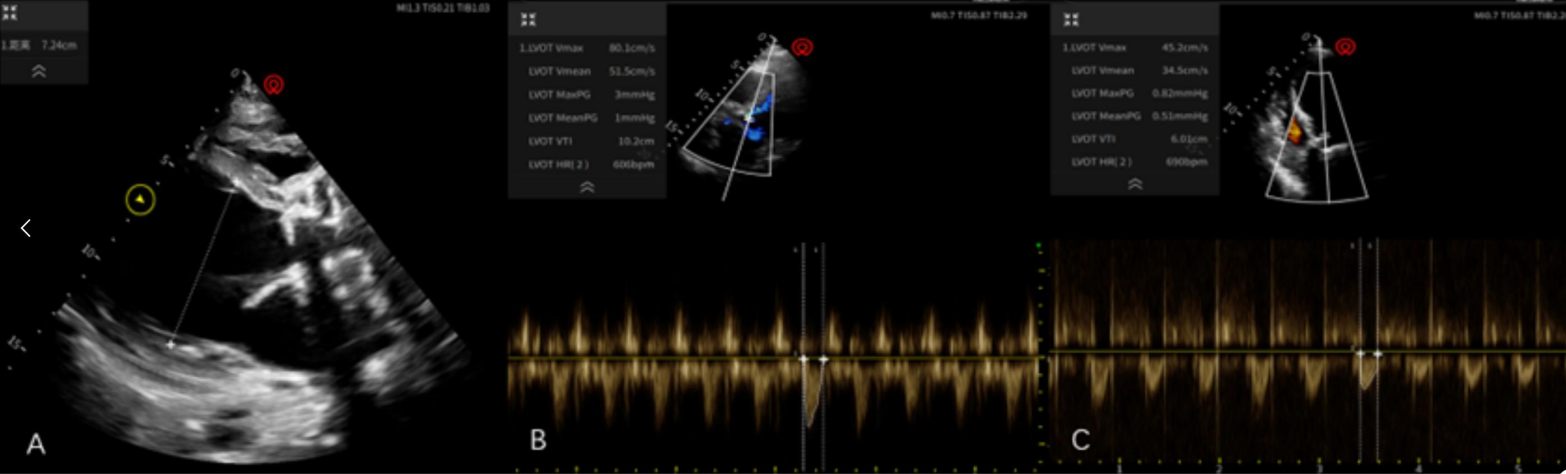
**(A)** Parasternal long-axis view shows diffusely weakened left ventricular contraction and severe left ventricular dilatation, with an anteroposterior left ventricular diameter of 7.2 cm; **(B)** aortic root velocity time integral (VTI) of 10 cm/s; **(C)** Aortic root velocity time integral (VTI) of 6 cm/s.

On the 8th day after admission, the patient only needed a small dose of vasoactive drugs to maintain blood pressure (NE: 0.08 ug/Kg/min, Ad: 0.011 ug/Kg/min). At this time, the HR was 102 bpm sinus rhythm, BP: 100/73 mmHg, and ECMO parameters were gradually reduced to 1,800 r/min, flow 2.0 L/min, and air flow 2.0 L/min. Bedside cardiac ultrasound was performed to prepare for the withdrawal of ECMO. In the parasternal long axis section, diffuse weakening of the left ventricle was seen, accompanied by severe dilatation of the left ventricle (left ventricular anteroposterior diameter was measured to be 7.2 cm), and the velocity time integral (VTI) of the aortic root was 10 cm/s. However, when the ECMO parameters were further reduced to 1,650 r/min and flow 1.5 L/min, BP dropped to 75/65 (68) mmHg, and the dose of vasoactive drugs needed to be increased, and tissue perfusion could not be met. At this time, echocardiography was performed again to measure the VTI, which had dropped to 6 cm/s (Figure 4), and we declared the weaning trial a failure (Figure 4). Two weeks after admission, we repeated the weaning trial, which still failed. We assessed the patient's inability to fully wean off ECMO support and recommended heart transplantation or LVAD replacement therapy. However, due to financial concerns, the family decided to forgo treatment. On the 15th hospital day, the patient requested removal of ECMO support and was declared clinically dead. Table 2 details the key events and timeline during the case hospitalization.

**Table 2:** Hospitalization timeline of the patient and the significant clinical events in the case.

Timeline	Timepoints	Significant clinical events
Day 0	03:00	The case was admitted to the hospital with chest pain for 3 days and was diagnosed with type A aortic dissection at an external hospital. He had respiratory rate of 18 bpm, heart rate of 85 bpm, blood pressure of 82/62 mmHg, as well as general fatigue and cold extremities. Vasoactive drugs were added to increase blood pressure.
	04:00	The case experienced a rapid drop in blood pressure, with a HR of 101 bpm and a BP of 70/54 mmHg. The NE dose was 1.11 μg/kg/min, and the patient was intubated and assisted with mechanical ventilation.
	05:00	The case had frequent ventricular tachycardia, and the electrocardiogram shows V1–V4 ST segment elevation, consistent with acute myocardial infarction.
	05:30	Abnormally elevated CK-MB and TNI; cardiac ultrasound showed diffuse weakening of the left ventricular wall and decreased left ventricular systolic function, further supporting the diagnosis of combined acute myocardial infarction and cardiogenic shock.
	08:30	He was taken to the operating room, at which time his heart rate was 112 bpm, blood pressure 79/50 mmHg, and SpO2 80%.
	16:25	The case surgery was completed, but he was unable to be taken off the extracorporeal circulation machine, so it was decided to leave the ECMO bridge to complete the surgery.
	19:00	The case was transferred to the ICU with ECMO, and the initial ECMO settings were 2,594 r/min, flow 3.45 L/min, and gas flow 3 L/min. At this time, NE: 0.9 ug/Kg/min and Ad: 0.3 ug/Kg/min were combined to maintain BP81/67 (72) mmHg.
Day 1	02:00	Blood pressure was monitored based on the right radial artery, with the goal of maintaining MBP >65 mmHg, and the dose of vasoactive drugs was gradually reduced.
	04:00	Disable the use of NE and Ad.
	06:00	The case blood gas analysis showed persistent metabolic acidosis with hyperlactatemia, suggesting tissue hypoperfusion. Blood pressure monitoring was reestablished on the left radial artery, and nephropathy and adrenocortical therapy were restarted. CRRT was connected in parallel to the ECMO circuit to improve the internal environment.
Day 2	08:00	The case continued to rely on NE: 0.84 ug/Kg/min, Ad: 0.25 ug/Kg/min to maintain blood pressure, and reported abnormally elevated inflammatory indicators. The patient was assessed to be in hypotonic shock, and antibiotic treatment was upgraded.
Day 3	08:00	The case hemoglobin remained at a low level despite intermittent infusion, leading to hyperbilirubinemia characterized by elevated indirect bilirubin. Hemolysis was suspected, and the flow rate was immediately optimized and the centrifugal pump speed was reduced to protect red blood cells.
Day 8	08:00	After lowering ECMO parameters, blood pressure dropped, requiring an increase in the dose of vasoactive drugs, but tissue perfusion could not be maintained, leading to failure of the weaning trial.
Day 14	08:00	Repeated weaning trials failed.
Day 15	10:39	Due to financial problems, the family did not consider heart transplantation and LVAD assistance and decided to abandon ECMO support and declare clinical death.

## Discussion

Marfan syndrome (MFS) is an autosomal dominant connective tissue disease. Its root cause is a mutation in the Fibrillin-1 gene, which leads to abnormalities in the structure and function of elastic fibers in the aortic wall. MFS is mostly familial, and about 50% of the patients' offspring will also be affected, and the disease shows significant variability (1). In this case, the patient's mother and sister both suffered from MFS, and both he and his mother had complications of AD. MFS is often accompanied by progressive dilatation of the aorta, forming aneurysms, and significantly increasing the risk of aortic dissection (AD). When AD affects the coronary arteries, it can lead to stenosis or occlusion of the coronary artery opening, causing acute myocardial infarction (AMI). Especially when the left coronary artery is affected, it can cause large-area myocardial ischemia and necrosis, which seriously threatens the patient's life. For patients with MFS combined with AD and left coronary artery occlusion causing AMI, treatment is extremely challenging. On the one hand, urgent restoration of coronary blood flow is required. On the other hand, for AD, appropriate treatment options should be selected based on the type of dissection and the patient's specific situation. For Stanford A-type interlayer, surgical treatment is often used, including ascending aorta replacement and Bentall procedure, but the surgery is very invasive and has a high complication rate (2, 3).

In this case, the patient suffered from an intramural hematoma at the aortic root compressing the left coronary artery, which directly led to extensive anterior wall acute myocardial infarction, resulting in severe cardiogenic shock and frequent malignant arrhythmias. However, prolonged myocardial ischemia and damage led to severe left ventricular systolic dysfunction. Although surgery involved grafting the left coronary artery with the internal mammary artery, restoring coronary blood flow, the recovery of cardiac contractile function was not ideal, and severe cardiogenic shock persisted. We therefore used ECMO to bridge the cardiopulmonary bypass (CPB) and conclude the surgery.ECMO partially or completely replaces the heart's pumping function, reduces the heart's workload, and creates a valuable “time window” for the recovery of damaged myocardium (4). The role of ECMO in the treatment of cardiogenic shock (PCS) after cardiac surgery has been recognized. The mortality rate of this type of untreated refractory shock is close to 100%. Globally, the number of cases of PCS treated with VA-ECMO has increased significantly (5). Gouveia D reported 13 patients with refractory cardiogenic shock after cardiac surgery who received intubation and veno-venous extracorporeal membrane oxygenation (VV-CMO) support (median time 6 days, range 1–16 days). Seven patients (53.8%) were successfully weaned from the ventricular membrane oxygenation (VEO). Two of them died of stroke and septic shock, respectively. One patient is still recovering in the intensive care unit, and four patients were discharged alive. The results of this study showed that approximately one-third of patients with refractory cardiogenic shock after surgery can survive after receiving VV-ECMO treatment (6).

There are generally three methods for arterial cannulation during ECMO bridging after PCS: femoral artery cannulation, axillary artery cannulation, and central cannulation. The femoral artery approach is usually the most commonly used due to its speed and simplicity. Traditional femoral artery cannulation is widely used due to its convenient operation, but it has limitations such as increased left ventricular afterload associated with retrograde perfusion, North-South syndrome (NSS), and lower limb ischemia (7). Furthermore, retrograde blood flow from femoral artery cannulation during ECMO may increase aortic pressure, leading to increased aortic valve regurgitation and consequently increasing left ventricular afterload, this LV overload situation may also deteriorate into a life-threatening accumulation of interrelated cardiopulmonary complications, cumulating in pulmonary hemorrhage (8). An over-distended LV exposed to high myocardial stress, strain, work and oxygen consumption, as well as reduced coronary blood flow, will likely be unable to recover. Pulmonary edema may primarily occur as a consequence of high LV filling pressures, but systemic inflammation mediated by shock and impending multi-organ failure, as well as blood contact to artificial extracorporeal surfaces, may contribute. In this context, acute lung injury has been shown to significantly impact on prognosis in patients receiving VA ECMO, even after successful bridge-to-bridge therapy, to avoid these complications, the LV requires decompression (9). When this occurs echocardiographic evidence of minimal aortic valve opening, “smoke” in the left atrium, left ventricle, or aorta (indicating stasis of blood); reduced arterial pulse pressure; or edema on a chest x-ray (especially when occurring in clusters) require intervention to enhance blood flow through the heart.use of either an intra-aortic balloon counterpulsation pump (IABP) or an Impella device is associated with statistically better survival despite increased complications and that percutaneous ventricular assist device (pVAD) use is associated with more complications despite comparable survival with IABP (10). Although axillary access does not fully alleviate the increase in left ventricular afterload inherent to peripheral V-A ECMO, the antegrade flow it provides results in comparatively lower afterload than femoral retrograde perfusion, By fostering, laminar, antegrade perfusion, axillary cannulation may contribute to improved myocardial recovery and decreased pulmonary congestion, particularly in patients with residual or recovering left ventricular function (11). Although central cannulation can provide antegrade blood flow to the aorta and significantly reduce left ventricular afterload compared to axillary and femoral artery cannulation, its high risk of bleeding, cardiac compression, inability to close the sternum, severe infection, and the need for a second thoracotomy to remove the catheter prevent its widespread clinical use (12). In this case, we had some remaining artificial vascular graft material after performing aortic dissection surgery. The distal right axillary artery was exposed through a subclavian incision, and the artificial graft was sutured in an “end-to-side” fashion. The ECMO perfusion cannula was then inserted into the artificial graft and secured with silk sutures. Complete hemostasis was achieved, and the skin incision was closed. This modified axillary artery cannulation perfectly solved the problem of distal limb ischemia and necrosis caused by peripheral cannulation, and also achieved antegrade blood flow similar to central cannulation, avoiding a series of complications related to increased left ventricular afterload. This bridging method has rarely been reported, although Yang et al. have used it in lung transplantation (13). Therefore, we suggest that in patients experiencing severe cardiogenic shock after aortic dissection surgery requiring ECMO bridging, and provided that the axillary artery is confirmed to be supplied by the true lumen, this modified axillary artery cannulation technique using a vascular graft can be considered as an alternative cannulation strategy to avoid the serious complications associated with central cannulation. Absolute contraindications include active local infection or cutaneous lesions at the intended cannulation site, which carry a high risk of bacteremia and systemic seeding, and untreated aortic dissection involving the brachiocephalic artery should be considered an absolute contraindication due to the potential for false lumen perfusion. Axillary artery cannulation is associated with several limitations that warrant careful consideration. Surgical arterial exposure is technically more challenging than femoral access, typically requiring a deltopectoral or infraclavicular approach that may prolong cannulation time, particularly in urgent or hemodynamically unstable scenarios (14). In this case, this modified axillary artery cannula was used to achieve the simultaneous existence of peripheral cannula and antegrade blood flow, and no complications of limb necrosis and left ventricular dilatation occurred. However, it was also found that this modified axillary artery cannula would cause a difference of nearly 20 mmHg in the blood pressure between the left and right arteries. The possible reason is that the blood flow from the ECMO perfusion tube to the distal right radial artery enters the microcirculation, resulting in a sudden increase in vascular tension. This also reminds us to avoid right radial artery blood pressure monitoring when using this modified axillary artery cannula in the future.

As an important alternative to ECMO arterial access, the axillary artery has shown significant value in specific clinical scenarios due to its anatomical characteristics and physiological advantages. Its core advantages are as follows: 1. Optimizing hemodynamic patterns: achieving antegrade perfusion, thereby avoiding NSS. The core physiological advantage of axillary artery cannulation is that it reestablishes a physiological blood flow direction through antegrade perfusion of the aortic arch and brachiocephalic trunk, fundamentally solving the NSS caused by retrograde perfusion of the femoral artery (15). Chamogeorgakis et al. (16) conducted a retrospective analysis of 81 VA-ECMO patients who underwent axillary artery cannulation. The results showed that this method can completely avoid the occurrence of NSS, and the incidence of lower limb ischemia was significantly reduced compared with 166 patients with femoral artery cannulation (24.7% vs. 41.5%, *P* < 0.01), suggesting that antegrade perfusion plays a key role in optimizing systemic blood flow distribution. Ohira et al. (11) conducted a multicenter study on 371 patients with cardiogenic shock ECMO and further confirmed that the incidence of NSS in the axillary artery cannulation group was 0, significantly lower than the 12.3% in the femoral artery group (*P* < 0.001), and the cerebral oxygen saturation (near infrared spectroscopy NIRS monitoring) was continuously higher than that in the femoral artery group 72 h after surgery (78% ± 5% vs. 71% ± 6%, *P* < 0.05), confirming the protective effect of antegrade perfusion on cerebral oxygenation. 2. The risk of limb ischemia is significantly reduced. The main complication of femoral artery cannulation is lower limb ischemia (incidence 10%–70%), the mechanism of which includes obstruction of the femoral artery lumen by cannulation, distal hypoperfusion and vasospasm. Axillary artery cannulation avoids this risk in two ways: the third segment of the axillary artery (outside the pectoralis minor muscle) is preferred, which is far away from the main branches and can preserve the distal blood flow of the axillary artery through the “end-to-side anastomosis artificial blood vessel” technique; Radwan et al. (17) conducted a single-center study on 179 patients with postoperative cardiogenic shock and showed that the incidence of upper limb ischemia in the right axillary artery cannulation group was only 6.1%, which was much lower than the lower limb ischemia rate reported in the literature for femoral artery cannulation (15%–40%), and no case required fasciotomy or limb amputation. Cakici et al. (18) compared 148 ECMO patients (side branch transplantation vs. percutaneous cannulation) and found that the acute limb ischemia rate in the axillary artery side branch transplantation group was only 2.7%, significantly lower than the 5.3% in the percutaneous femoral artery group (*P* < 0.05), confirming the reliability of this technology in protecting limb blood flow. 3. Reduced infectious complications. Femoral artery cannulation is located near the groin area and requires long-term exposure, resulting in a local infection rate of up to 20%, significantly prolonging hospitalization time (19). The axillary artery is located in the subclavian/deltoid region, has a relatively clean anatomical position, and can be fixed through a subcutaneous tunnel, reducing the risk of contamination. Ohira et al. (20) studied 80 ECMO patients after heart transplantation and found that the cannulation-related wound infection rate in the axillary artery cannulation group was only 2.5%, significantly lower than the 15.0% in the femoral artery group (*P* < 0.01), and no case of sepsis was caused by infection. In addition, axillary artery cannulation does not require the sternum to be open (compared to central cannulation), which can avoid mediastinal infection (incidence <1% vs. 8%–12% for central cannulation) (21), further reducing the risk of systemic infection. 4. Improve brain and coronary artery perfusion: Enhance neuroprotection and myocardial recovery. Adequate oxygenation of the brain and coronary arteries is the core goal of reducing long-term neurological damage and promoting myocardial recovery during ECMO support. Axillary artery cannulation can significantly improve the perfusion quality of these key organs by optimizing hemodynamics. In a prospective study of 37 patients on peripheral VA-ECMO, Salna et al. (22) used transcranial Doppler (TCD) to monitor middle cerebral artery (MCA) blood flow and found that the mean MCA blood flow velocity in the axillary artery cannulation group (58 ± 8 cm/s) was significantly higher than that in the femoral artery group (42 ± 7 cm/s, *P* < 0.001), and the blood flow pulsatility index (PI) was lower (0.7 ± 0.1 vs. 1.1 ± 0.2, *P* < 0.001), suggesting that axillary artery perfusion can provide more stable and continuous cerebral blood flow and reduce cerebral ischemia-reperfusion injury. A blood flow simulation study by Feiger et al. (23) further confirmed that axillary artery cannulation can maintain carotid artery oxygen delivery >15 mL/(kg min) at an ECMO flow rate of 1–3 L/min, while femoral artery cannulation requires a flow rate >4.9 L/min to achieve the same level, which is particularly suitable for patients with low-flow ECMO support (such as those in the myocardial recovery period). Regarding coronary artery perfusion, antegrade blood flow in the axillary artery can directly increase aortic root pressure and coronary diastolic perfusion pressure. Sibut-Pinote et al. (24) compared axillary and femoral ECMO in a low cardiac output model and found that the coronary blood flow in the axillary artery group increased by 22% ± 5% compared with the femoral artery group (*P* < 0.05), and myocardial tissue oxygen partial pressure (PO_2_) was significantly increased (38 ± 4 mmHg vs. 29 ± 3 mmHg, *P* < 0.05), creating better conditions for the recovery of damaged myocardium. A prospective study by Andrei et al. (25) also showed that the velocity time integral (VTI) of the aortic root in patients with axillary artery ECMO increased with increasing ECMO flow (*r* = 0.82, *P* < 0.001), suggesting that left ventricular ejection function and ECMO blood flow showed a synergistic improvement trend, while the VTI of the femoral artery group decreased with increasing ECMO flow (*r* = −0.76, *P* < 0.001), indicating the risk of left ventricular outflow tract obstruction. Axillary artery cannulation for VA-ECMO is a safe and effective alternative to FA cannulation.

The definition of successful VA-ECMO weaning is usually that the patient survives for more than 48 h after ECMO removal. Some experts believe that after ECMO removal, the patient does not need to continue mechanical support within the next 30 days (26). According to a large number of literature reports, the proportion of patients who successfully wean from VA-ECMO ranges from 30% to 75% (27). Regarding VA-ECMO weaning, the success criteria usually include maintaining stable hemodynamics with low-flow support and a small dose of vasoactive drugs (such as mean arterial pressure >65 mmHg, pulse pressure >10 mmHg), and at the same time, cardiac ultrasound assessment shows significant recovery of cardiac function (such as left ventricular outflow tract velocity time integral >0.12 m/s, left ventricular ejection fraction ≥25%–30%) (28). Obviously, the above requirements were not met on the 8th and 14th days after surgery in this case, and the weaning was declared a failure. Some scholars have conducted predictors of weaning failure in case of VA-ECMO implantation. Multivariate analysis revealed three predictors of weaning failure: a post-test SBP ≤120 mmHg, a delay in ECMO weaning >7 days and a history of ischemic heart disease. LVEF can also be taken into account with a pre-test LVEF ≤25% and/or a post-test LVEF ≤40% (*p* = 0.052) (29). Whether there is acute renal damage during ECMO bypass and the need for CRRT is also an important reference indicator for the patient's prognosis (30). In most cases, VA-ECMO is intended to serve as a bridge to recovery. When recovery is not possible and the patient remains dependent on VA ECMO, the prognosis is poor or depends on long-term treatment options, with the only possible solution being the implantation of a mechanical circulatory support (MCS) device, i.e., an LVAD or biventricular assist device (BIVAD), or emergency heart transplantation (31).

## Summarize

This case report a 19-year-old male with Marfan syndrome and Stanford type A aortic dissection who developed acute myocardial infarction and cardiogenic shock due to coronary artery compression caused by hematoma. We employed a modified axillary artery cannulation for VA-ECMO bridging. This cannulation technique offers advantages such as optimized hemodynamics, significantly reduced risk of limb ischemia, fewer infectious complications, and improved cerebral and coronary perfusion, making it one of the more ideal ECMO cannulation strategies. However, cardiac function never recovered in this case, and after two failed ECMO weaning trials, the family abandoned LVAD or heart transplantation due to financial constraints. The patient died on hospital day 15.

## Data Availability

The original contributions presented in the study are included in the article/Supplementary Material, further inquiries can be directed to the corresponding author.
